# Functionalization of Polycaprolactone 3D Scaffolds with Hyaluronic Acid Glycine-Peptide Conjugates and Endothelial Cell Adhesion

**DOI:** 10.1021/acs.biomac.4c01559

**Published:** 2025-02-24

**Authors:** Tamilselvan Mohan, Fazilet Gürer, Doris Bračič, Florian Lackner, Chandran Nagaraj, Uroš Maver, Lidija Gradišnik, Matjaž Finšgar, Rupert Kargl, Karin Stana Kleinschek

**Affiliations:** † Graz University of Technology, Institute of Chemistry and Technology of Biobased System, Stremayrgasse 9, 8010 Graz, Austria; ‡ University of Maribor, Faculty of Mechanical Engineering, Laboratory for Characterisation and Processing of Polymers, Smetanova ulica17, 2000 Maribor, Slovenia; § University of Maribor, Faculty of Medicine, Institute of Biomedical Sciences, Taborska Ulica 8, 2000 Maribor, Slovenia; ∥ Department of Internal Medicine, Division of Pulmonology, Medical University of Graz, 8010 Graz, Austria; ⊥ University of Maribor, Faculty of Chemistry and Chemical Engineering, Laboratory for Analytical Chemistry and Industrial Analysis, Smetanova ulica 17, 2000 Maribor, Slovenia; # University of Maribor, Institute of Automation, Faculty of Electrical Engineering and Computer Science, Koroska cesta 46, 2000 Maribor, Slovenia; ∇ Members of the European Polysaccharide Network of Excellence (EPNOE), Celestijnenlaan 200 F, 3001 Leuven, Belgium

## Abstract

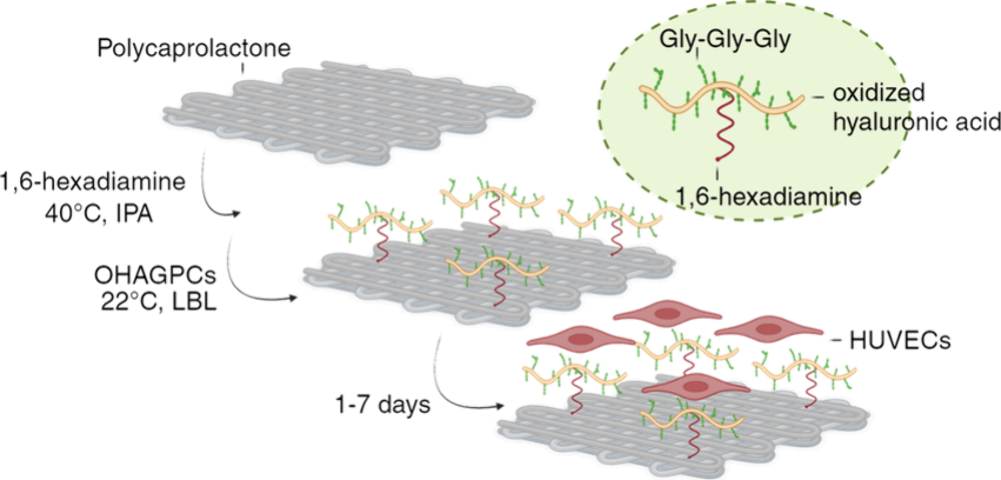

This study enhances the bioactivity of polycaprolactone (PCL) scaffolds for tissue engineering by functionalizing them with oxidized hyaluronic acid glycine-peptide conjugates to improve endothelial cell adhesion and growth. Hyaluronic acid was conjugated with a glycine-peptide to create a bioactive interface on PCL (static water contact angle, SCA(H_2_O): 98°). The scaffolds were fabricated using a melt extrusion 3D printing technique. The HA-glycine peptide conjugates were oxidized and immobilized on aminolyzed PCL via Schiff-base chemistry, introducing hydrophilicity (SCA(H_2_O): 21°), multiple functional groups, and a negative zeta potential (−12.04 mV at pH 7.4). A quartz crystal microbalance confirmed chemical conjugation and quantified the mass (8.5–10.3 mg m^–2^) of oxidized HA-glycine on PCL. The functionalized scaffolds showed enhanced swelling, improved mechanical properties (2-fold increase in strength, from 26 to 51 MPa), and maintained integrity during degradation. In-vitro experiments demonstrated improved endothelial cell adhesion, proliferation and viability, suggesting the potential for vascularized tissue constructs.

## 1 Introduction

Tissue engineering (TE) has emerged as a promising approach for regeneration and repair of damaged tissues and organs.^1,2^ A key challenge in this field is creating scaffolds that not only provide a structural framework for cell attachment and proliferation but also closely mimic the natural extracellular matrix (ECM) to promote tissue regeneration.^3,4^ The advent of 3D printing technology has revolutionized scaffold fabrication, offering unprecedented precision and customization in scaffold design, which is crucial for meeting the specific requirements of different tissues.^5−7^ Among the various materials utilized for scaffold fabrication, polycaprolactone (PCL) stands out due to its ability to interact with preserving its physicochemical property and degradability, and mechanical properties, making it an excellent candidate for 3D printing applications, particularly for small-diameter vascular grafts (SDVGs, <6 mm).^8−11^ PCL’s slow degradation rate makes it suitable for applications where long-term scaffold stability is required. However, despite these advantages, PCL scaffolds often face limitations related to bioactivity, hydrophobicity, particularly in promoting endothelial cell adhesion and proliferation.^12,13^ This necessitates modifications to enhance their biological performance, making them more conducive for SDVGs.^8^ Extensive efforts have been made to modify the PCL scaffold surface to increase hydrophilicity, mainly through covalent chemical conjugation and noncovalent physical adsorption methods.^14,15^ In physical adsorption via van der Waals interactions,^16^ adhesion proteins such as fibrinogen, fibronectin,^17^ and other ECM proteins are commonly used to create hydrophilic surfaces.^18,19^ However, these methods often suffer from long-term stability due to reverse dissociation of the bound proteins. In contrast, chemical conjugation via the coupling of carboxylic acid group (COOH), amino group (NH_2_),^20,21^ alcohol group (OH),^22^ click chemistry, aminolysis,^14,21^ and sulfhydryl-maleimide coupling^23^ have been often frequently used to covalently attach bioactive and hydrophilic functional groups to the surface of PCL. Additionally techniques, such as oxygen plasma treatment,^24^ chemical etching, or γ-ray irradiation have also been used to introduce biomolecules such as proteins, peptides, or growth factors on the surface.^25^ Compared to other chemical treatments, aminolysis offers a combination of mild reaction conditions, precise surface modification and reactive amino groups, making it a highly suitable for further functionalization. Moreover, the covalent chemical conjugation methods ensure stable surface modification.^15^

Surface modification using polysaccharides such as chitosan,^26^ carboxymethyl cellulose^27,28^ or alginate,^29^ via chemical conjugation, has emerged as a promising strategy to enhance interaction with microcellular environment.^30−32^ Among other polysaccharides, hyaluronic acid (HA) stands out as a natural and abundant component of the extracellular matrix (ECM). Its remarkable capacity to interact with cells and its biodegradability have made it a focal point of interest in TE.^33−37^ HA’s hydrophilic nature and ability to form a hydrated matrix create an ideal environment for cell growth and proliferation. HA has been conjugated with various peptides to create biofunctionalized scaffolds with enhanced cell interactions.^38−41^ Peptides derived from ECM proteins, growth factors, or cell-adhesion motifs can mimic specific cell-ECM interactions and modulate cellular behavior, making them another important class of bioactive molecules in TE.^39,41,42^ Chemical modification of HA with bioactive peptides can further enhance its functionality and tailor its biological properties for specific applications.^43^ Until now, several ECM protein-based peptides and peptide derivatives have been evaluated for cell adhesion ability, including RGD (Arg-Gly-Asp), IKVAV (Iso-Lys-Val-Ala-Val), YIGSR (Tyr-Iso-Gly-Ser-Arg), and DGEA (Asp-Gly-Glu-Ala).^44−46^ Additionally, researchers have investigated the cell adhesion abilities of synthetic peptides^47^ such as (Pro-Pro-Gly)_8_, (Gluv-Pro-Arg-Gly-Asp-Thr) and (Pro-Hyp-Gly)_8._ However, applications of such synthetic RGD or RGD derivatives can be expensive due to their intricate designing, synthesis and purification process.^48^ Therefore, researchers continue to explore cost-effective cell adhesion motifs to establish strong adhesion at cell–substrate interfaces in TE.

In this study, we selected glycine/glycine peptides to chemically modify HA as model components for expensive ECM cell adhesion peptides and their peptide derivatives. Glycine was selected due to its abundance in cell adhesion motifs. Previous studies have demonstrated that glycine peptide conjugates enhance cell adhesion and spreading by promoting integrin-mediated signaling pathways.^48,49^ In our earlier research, we successfully conjugated and characterized glycine and glycine peptides to HA via carbodiimide chemistry.^39^ Despite significant advances in polycaprolactone (PCL) scaffold functionalization, there is a notable lack of research on the chemical functionalization of PCL with HA-glycine amino acids and glycine peptide conjugates, as well as their effects on endothelial cell (EC) adhesion and growth. Therefore, this study focuses on the chemical conjugation of HA-glycine peptide conjugates to 3D-printed PCL scaffolds to create a biomimetic microenvironment that enhances cell-material interactions, particularly with ECs. Unlike conventional ECM peptides that are often expensive and complex to synthesize, this study introduces glycine/glycine peptides as a cost-effective and biologically relevant alternative to promote cell adhesion. This is the first report to utilize HA-glycine peptide conjugates for scaffold functionalization, filling a significant gap in research on PCL modification.

In TE, the mechanical properties of scaffolds are essential in designing and fabricating structures tailored for specific tissue regeneration applications, as different tissues have distinct mechanical characteristics, such as stiffness, elasticity, and strength.^50^ For instance, soft tissues like skin and cartilage require scaffolds with higher elasticity to support their functional roles,^51^ whereas scaffolds intended for bone tissue regeneration must exhibit greater stiffness to provide sufficient support for weight-bearing. Consequently, selecting appropriate mechanical properties for scaffolds is critical to ensure they offer the necessary structural support and foster effective tissue growth. Additionally, the mechanical properties significantly influence cellular behavior within the scaffold, as well as its integration and stability within the host tissue.^51^

To incorporate all the above-mentioned properties into the PCL, the 3D-printed PCL scaffolds were first functionalized with amino groups using 1,6-hexanediamine via aminolysis. Subsequently, the HA-glycine and HA-glycine peptide conjugates were oxidized with sodium periodate and attached to the aminolyzed PCL scaffolds through Schiff-base chemistry. The functionalized scaffolds were then characterized for their chemical composition, morphology, wettability, surface charge, and mechanical properties. The suitability of these scaffolds for vascular TE was evaluated by assessing the viability and proliferation of human umbilical vein endothelial cells (HUVECs). HUVECs were chosen for this study since they commonly used in vascular TE due to their physiological relevance, ease of availability, and their ability to mimic endothelial behavior, such as proliferation and angiogenesis. They also provide a standardized model for reproducibility and comparison with other studies.^52^ Functionalized 3D-printed polycaprolactone (PCL) scaffolds with oxidized hyaluronic acid (HA) glycine-peptide conjugates offer innovative solutions for vascularized tissue engineering. By enhancing endothelialization, bioactivity, and mechanical integrity, they address key challenges in vascular regeneration. Combined with bioprinting and patient-derived cells, these scaffolds could enable personalized tissue constructs, advancing regenerative medicine and organ repair.

## 2 Experimental Section

### 2.1 Materials

Polycaprolactone (*M*_w_: 45 kDa), glycine methyl ester hydrochloride (Gly-OMe), 4-(4,6-Dimethoxy-1,3,5-triazin-2-yl)-4-methylmorpholinium chloride (DMT), sodium metaperiodate (NaIO_4_), sodium chloride (NaCl), methanol hydrochloride (1.25 M), dimethyl sulfoxide (DMSO), isopropanol, 1,6-hexanediamine, deuterium oxide (D_2_O, 99.9%), ninhydrin, sodium acetate and acetic acid were purchased from Sigma-Aldrich, Austria. Sodium salts of hyaluronic acid (HA, Mw: ∼90 kDa), diglycine methyl ester (DiGly-OMe) and triglycine ethyl ester (TriGly-OEt) were purchased form Carbosynth, United Kingdom. Dialysis membrane (Mw cutoff: 12 kDa) was purchased Roth chemicals, Austria. Milli-Q water from a Millipore (MA, USA) water purification system (resistivity ≥18.2 MΩ cm, pH 6.8) was used for the preparation of all aqueous solutions during these experiments.

Human umbilical vein endothelial cells (HUVEC) (1 × 10^6^ cells), medium 200, low serum growth supplement, live/dead assay, advanced Dulbecco’s Modified Eagle Medium (ADMEM) were purchased from Thermo Fisher Scientific, Austria. 3-(4,5-dimethylthiazol-2-yl)-5-(3-carboxymethoxyphenyl)-2-(4-sulfophenyl)-2H-tetrazolium (MTS) dye was purchased from Promega, Germany. Heat inactivated fetal bovine serum was acquired from Gibco (by Thermo-Fisher Scientific) Waltham, Massachussets, USA. Penicillin, streptomycin, l-glutamine and trypsin-ethylenediaminetetraacetic acid (EDTA) were purchased from Sigma-Aldrich, Merck KGaA, Darmstadt, Germany. iFluor 555 Reagent (ab176756, CytoPainter Phalloidin) and mounting medium with DAPI (ab104139) were purchased from Abcam plc, Austria. Fixation solution was purchased from Merck, Millipore.

#### 2.1.1 Synthesis of Hyaluronic Acid Glycine-Peptide Conjugates

The conjugation of Gly-OMe, DiGly-OMe and TriGly-OEt to HA proceeded as follows: First, HA (3.2 mmol COONa, 1.287 g) was dissolved in 100 mL Milli-Q water and stirred overnight. To this HA solution, DMT (3.2 mmol, 0.886 g) and Gly-OMe (16 mmol, 2.01 g), or DiGly-OMe (16 mmol, 2.92 g), or TriGly-OEt (16 mmol, 4.06 g) were added. This mixture was allowed to react for 24 h at room temperature and at constant stirring. Afterward, the reaction mixture was dialyzed against a 2 M NaCl solution for 1 day, followed by dialysis against Milli-Q water for 3 days. The mixture was then frozen at −37 °C for 2 days and subsequently lyophilized at 25 °C and 103 mbar for 2 days. The molar ratio of the reaction between HA, DMT and glycine/glycine-peptides was kept at 1:1:5.

#### 2.1.2 Oxidation of Hyaluronic Acid Glycine-Peptide Conjugates

The oxidation of HA-glycine and HA glycine-peptide conjugates was performed following a protocol outlined in the literature.^53^ In brief, 100 mg of the purified and isolated conjugates from section 2.1.1 were dissolved in 20 mL of Milli-Q water. Then, 400 μL of 0.5 mmol NaIO_4_ was added to the solution, thoroughly mixed at room temperature for 1 h away from light. To terminate the reaction, 100 mmol of ethylene glycol was added to the mixture. Afterward, the resulting solutions were dialyzed with a dialysis membrane (MW: 12 kDa) against a 2 M NaCl solution for 1 day, followed by dialysis against Milli-Q water for 3 days. The solutions were then frozen for 2 days at −37 °C, followed by lyophilization for 2 days at −25 °C under a pressure of 10^–3^ mbar.

#### 2.1.3 Quantification of Aldehyde Groups

The Ninhydrin assay was used to quantify the formation of aldehyde groups in the oxidized hyaluronic acid glycine/glycine peptide conjugates. Since aldehyde groups do not directly react with ninhydrin, an excess of glycine was added to the conjugates. The unreacted glycine was then measured to determine the degree of oxidation. In summary, solutions of 0.1 mg mL^–1^ conjugates and 0.01 M glycine were prepared in carbonate buffer (pH 8.5). Subsequently, 1 mL of the conjugate solution was combined with 1 mL of glycine and incubated for 3 h on a shaker at room temperature. Following this, 2 mL of ninhydrin solution was added, and the mixture was incubated at 100 °C for 15 min. Finally, the solutions were transferred into UV cuvettes, and the number of amino groups was assessed by measuring the optical density (absorbance) at a wavelength of 570 nm.

#### 2.1.4 3D Printing of PCL Scaffold

The PCL scaffolds were prepared using a 3D BioScaffolder 3.1 (GeSIM, Germany). A 10 mL metal syringe (GeSIM, Germany) with an inner nozzle diameter of 250 μm was used to extrude PCL onto a polystyrene Petri dish (diameter: 5 cm) or into 96-well plates. The syringe was densely filled with PCL pellets and heated to 90 °C for 1 h (before printing). Circular scaffolds (radius: 5 mm, height: 0.5 mm) were printed layer-by-layer. These dimensions were created using GeSIM Robotics BS3.1/3.2 software. For printing scaffolds, the dispensing pressure was set from 320 to 350 kPa and the distance between the strands was set to 200 μm. The print patterns of each subsequent layer were rotated by 90° and the printing speed was 3 mm/s. For the tensile tests, Dog bone-shaped tensile test specimens were 3D printed according to DIN 53504 S3A using the same parameters as described above.

#### 2.1.5 Aminolysis of 3D-Printed PCL Scaffold and Quantification of Amino Groups

The protocol for aminolysis of 3D-printed PCL scaffolds was adapted from the literature.^54^ Initially, the PCL scaffolds (diameter: 9.5 mm) were washed several times with Milli-Q water. Subsequently, they were immersed in a solution of 0.43 mol L^–1^ 1,6-hexanediamine (dissolved in isopropanol) for 120 min at 37 °C. Afterward, the scaffolds were thoroughly rinsed with Milli-Q water to remove any unreacted or residual 1,6-hexanediamine. The resulting aminolyzed samples were designated as PCL-A. To determine the amount of newly introduced amino groups on the PCL scaffolds, the ninhydrin assay was used.^55^ Each sample was immersed in 2 mL of 0.1 mol L^–1^ ninhydrin/ethanol solution and heated to 80 °C for 15 min. Subsequently, 8 mL of 1,4-dioxane was added into the ninhydrin solution to dissolve the PCL. Finally, the solutions were transferred into UV-cuvettes, and the concentration of amino groups was determined by measuring the optical density (absorbance) at a wavelength of 538 nm.

#### 2.1.6 Conjugation of Oxidized Hyaluronic Acid Glycine/Glycine Peptide Conjugates (OHAGPCs) to Aminolyzed PCL Scaffolds

The Schiff-base chemistry was used to chemically immobilize the oxidized conjugates of HA-glycine and HA-glycine peptide onto the PCL-A scaffolds and PCL-A thin solid films.^56^ To enhance the immobilization, a layer-by-layer deposition method was used. Solutions of the conjugates (0.1 mg mL^–1^) were initially prepared in carbonate buffer (pH 8.5). The PCL-A scaffold was immersed in the conjugate solution for 3 h at room temperature. Subsequently, the scaffolds were rinsed with Milli-Q-water five times and dried in a vacuum oven at 40 °C for 30 min. This procedure was repeated three times.

##### 2.1.6.1 Preparation of Polycaprolactone (PCL) Thin Films and QCM-D Studies

Thin films of polycaprolactone (PCL) were prepared on Au-sensors for QCM-D studies. The Au-coated sensors were cleaned prior to film preparation: The sensors were initially soaked into a mixture of H_2_O/H_2_O_2_ (30 wt %)/NH_4_OH (5:1:1; v/v/v) for 10 min at 70 °C. Afterward, they were immersed in “piranha” solution (H_2_O_2_ (30 wt %)/H_2_SO_4_ (98 wt %) (1:3; v/v)) for 60 s, rinsed with water, and blow-dried with N_2_ gas. For spin coating on Au-sensors, 70 μL of the PCL solution (1 wt %, dissolved in chloroform) was used. The films were spin coated at a spinning speed of 4000 rpm, an acceleration of 2500 rpm s^–1^ for 60 s using a Polos MCD wafer spinner (APT corporation, Germany).^10,17^ All PCL substrates were dried with N_2_ gas and stored under ambient conditions until further use.

The PCL-A coated gold crystals were mounted in the QCM-D flow cell and equilibrated with Milli-Q water and a carbonate buffer (pH 8.5) until a stable resonance frequency was achieved. Subsequently, OHA-glycine/glycine peptide conjugates (0.2 wt %, dissolved in 0.1 M carbonate buffer) were pumped over the PCL-A film for 90 min. This was followed by a rinse with carbonate buffer and then with water (pH 7) for an additional 30 min.

### 2.2 Analytical Methods

#### 2.2.1 Wettability

To evaluate changes in the wettability of PCL scaffolds after OHA-glycine or glycine-peptide conjugation, static water contact angle (SWCA) measurements were conducted using an OCA15+ contact angle measurement system (Dataphysics, Germany). The measurements were performed at room temperature using Milli-Q water, with a drop volume of 3 μL. A minimum of two independent 3D-printed scaffolds were analyzed both before and after conjugation to ensure reliability. For stability test, all scaffolds (uncoated and coated/conjugated) were stored in cell culture medium for 7 days, rinsed with PBS buffer and Milli-Q water, then dried using nitrogen gas. Each SWCA value represented the average of at least five drops of liquid per surface.

#### 2.2.2 Zeta Potential Measurements

Zeta potential assessments of both PCL-A and PCL scaffolds were carried out using SurPASS 3 (Anton Paar GmbH, Austria) with the adjustable gap cell D15. Samples, each with a diameter of 15 mm, were affixed to the sample holder using double-sided adhesive tape. The distance between sample surfaces was set at 100 ± 10 μm. The electrolyte solution concentration was maintained at 1 mM KCl, and the pH was automatically adjusted with either 0.05 M NaOH or 0.05 M HCl. Zeta potential pH dependence was evaluated within the pH range of 3–10. A pressure gradient of 20 to 600 mbar was applied to induce the streaming potential.

#### 2.2.3 Quartz Crystal Microbalance with Dissipation (QCM-D)

A QCM-D instrument (model E4) from Q-Sense (Gothenburg, Sweden) was used to monitor the conjugation and adsorbed mass of OHA-glycine/-glycine peptides to PCL-A thin films.^10,17,57,58^ The instrument simultaneously measures changes in the resonance frequency (Δ*f*) and energy dissipation (Δ*D*) of the oscillating piezoelectric crystal arising from the changes in its mass and elasticity upon adsorption or desorption of components and ions from the aqueous solutions. If the elasticity of the adsorbed coatings insignificantly differs from that of the crystal, the mass of adsorbed coatings can be related to Δ*f* by the Sauerbrey equation as follows:
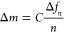
1where Δ*f*_*n*_ is the observed frequency shift, *C* is the Sauerbrey constant (−0.177 mg Hz^1–^ m^–2^ for a 5 MHz crystal), *n* is the overtone number (*n* = 1, 3, 5, etc.), and Δ*m* is the change in mass of the crystal.

The dissipation energy (*D*) refers to the frictional losses that lead to damping of the oscillation depending on the elastic properties of the material. *D* is defined as
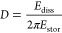
2where *E*_diss_ is the energy dissipated and *E*_stor_ is the total energy stored in the oscillator during one oscillation cycle.

#### 2.2.4 X-ray Photoelectron Spectroscopy (XPS)

XPS analysis was performed using the AXIS Supra+ device (Kratos Analytical Ltd., Manchester, UK) equipped with an Al K_α_ source and a monochromator. The analysis spot size had a radius of 110 μm. The measurements were performed at a 90° takeoff angle. The binding energy (EB) scale was corrected using the C–C/C-H peak in C 1s spectra that was centered at 284.8 eV. Spectra acquisition was performed using a hemispherical analyzer and a pass energy of 160 eV for the survey spectra and 40 eV for the high-resolution spectra. Sputtering was performed with a 10 keV Ar_2000_^+^ gas cluster ion beam (GCIB), with a raster size of 2 mm by 2 mm. XPS data were measured and processed using ESCApe 1.4 software (Kratos Analytical Ltd., Manchester, UK).

#### 2.2.5 Analysis of Swelling Capacity and Weight Loss

The swelling kinetics of the scaffolds in biofluid (ADMEM + 5% FBS + 100 IU mL^–1^ penicillin and 0.1 mg mL^–1^ streptomycin) were investigated using a gravimetric method.^7,59,60^ The cylinder-shaped scaffolds (*r* = 7 mm, *h* = 5 mm) were weighed (initial weight, *W*_0_), immersed in 5 mL biofluid (pH 7.4) at 37 °C. At predetermined time intervals (*W*_t_), the scaffolds were removed from the biofluid, wiped dry carefully by a filter paper only on the surface and weighed again. The swelling capacity at time *t* was calculated using Equation 1.
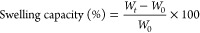
3

To determine the weight loss upon contact with bioliquid, the scaffolds (initial weight, *W*_0_) were placed in a beaker with 5 mL of biofluid at 37 °C and stirred at 200 rpm. At predetermined intervals (0 h to 4 weeks), the scaffolds were removed from the biofluid, washed three times with water and lyophilized as mentioned above. The remaining weight (RW) of the scaffolds was calculated as follows:
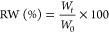
4

#### 2.2.6 Mechanical Testing

Special specimens with Dog bone shape for mechanical testing were prepared using 3D printing. Scaffolds were tested in a Universal Testing Machine (Zwick Roell, Type BT1-FB010TN.D30, Ulm, Germany) equipped with a 5 KN load cell was used to perform tensile a with a speed of 50 mm min^–1^ and standard clamps. All experiments were performed in quadruplicates. The experiments were performed according to DIN 53504 S3A. Scaffolds were immersed in 0.1 M PBS buffer at 37 °C for 24 h prior to tensile testing to evaluate the influence of swelling on their mechanical properties. The sample dimensions before and after coating and storage in solution were carefully using digital caliper and no dimensional changes after were found. The stress–strain curves/data were normalized using the actual cross-sectional area and volume of the samples postcoating and swelling. This ensured that changes in mechanical properties were attributed to the coating, not dimensional alterations. For scaffolds degraded in the presence of ADMEM up to 56 days (8 weeks), tensile testing was conducted under the same conditions as those used for scaffolds immersed in PBS.

### 2.3 In-Vitro Analysis on HUVEC Cells

#### 2.3.1 Cell Culture and Preparation for Analysis

HUVECs were cultured in Advanced DMEM supplemented with 5% FBS, 100 IU/mL penicillin, 0.1 mg mL^–1^ streptomycin, and 2 mM l-glutamine. Throughout the experiment, the medium was refreshed every 3 days. Subsequently, the samples underwent sterilization using 70% ethanol, followed by rinsing in PBS before placement in P24 microtiter plate wells. The samples were then dried in a microbiological cabinet for 12 to 18 h. Following this, 10,000 cells (50 μL) were seeded onto the materials and allowed to attach to the substrate during a 4-h incubation period at 37 °C. The culture was supplemented with 500 μL of Advanced DMEM media containing 5% FBS, 100 IU/mL penicillin, 0.1 mg mL^–1^ streptomycin, and 2 mM l-glutamine. Cell-free samples were included as controls. Assessments, including MTS, Live/Dead, and Phalloidin staining assays, were conducted at four different time points during the experiment: 24 h, 48 h, 72 h, and 7 days.

#### 2.3.2 The MTS Cell Viability Assay

The experiments were performed using cell-free media supplemented with 5% FBS. Both samples and controls were treated with 20% MTS reagent and then incubated for 3 h in a controlled environment, where the incubator maintained at 37 °C and 5% CO_2_. Following incubation, 100 μL of medium was transferred into a P96 microtiter plate, and the absorbance at 490 nm was measured with Varioskan Flash microplate reader (Thermo Fisher Scientific, ZDA). The experiment was conducted in triplicate for each sample at every time point, and the entire set of experiments was independently repeated on three separate occasions. These independent replicates were performed to ensure that the observed effects are reproducible and not influenced by random chance events.

#### 2.3.3 The Live/Dead Test

The Live/Dead fluorescence staining was conducted in triplicate for all materials at each time point. Results were compared between samples with cells and those without cells. The staining procedure involved the following steps: First, the cell culture medium (Advanced DMEM) was aspirated, and the scaffolds were washed once with PBS. Subsequently, the live/dead assay dye (Biotium) was added to the well plates, and the scaffolds were incubated in darkness at ambient conditions for 30 min to assess HUVECs viability. Following incubation, the samples were transferred to new wells filled with 500 μL PBS each. Imaging of the cells was performed using an EVOS FL fluorescence microscope (Thermo Fisher Scientific, Waltham, Massachusetts, USA) at a magnification of 10×.

#### 2.3.4 The Immunofluorescence Staining with Phalloidin

For visualizing cell adhesion and morphology on OHAGPCs conjugated samples, cells were stained with phalloidin dye (iFluor 555 Reagent) at each time point. Initially, the medium was aspirated, and the scaffolds were washed with PBS to eliminate any residual contaminants. Cells were then fixed using a cell fixation solution (diluted 1:5 in Milli-Q water) at room temperature for 20 min before staining. Following fixation, they were rinsed three times with cold PBS for 5 min each, after which Phalloidin was applied. Subsequently, the samples were incubated for 90 min in darkness at ambient conditions. After incubation, the scaffolds were rinsed twice with PBS and once with Milli-Q water. Two drops of mounting medium were then applied and allowed to polymerize for 5 min before the samples were inverted onto a glass slide. Finally, the samples were examined under a 10× magnification EVOS FL fluorescent microscope (Thermo Fisher Scientific, Waltham, Massachusetts, USA).

#### 2.3.5 Statistical Analysis

All numerical values are presented as mean ± SEM. Statistical analysis was performed using Prism 8.4.3 (GraphPad, San Diego, CA, USA). Statistical analyses were performed on pooled data from all independent replicates. For comparisons between groups, [specific Dunnett test, e.g., one-way ANOVA test] was used, and significance was determined at a threshold of *p* < 0.05. The Dunnett test confirmed that all experimental data followed a normal distribution, justifying the use of one-way ANOVA for analysis. Statistical significance was set at *P* < 0.05. Significant differences compared to the control sample (PCL) are indicated with an asterisk (*).

## 3 Results and Discussion

### 3.1 Characterization of Hyaluronic Acid Glycine and Glycine Peptide Conjugates

**Figure 1:**
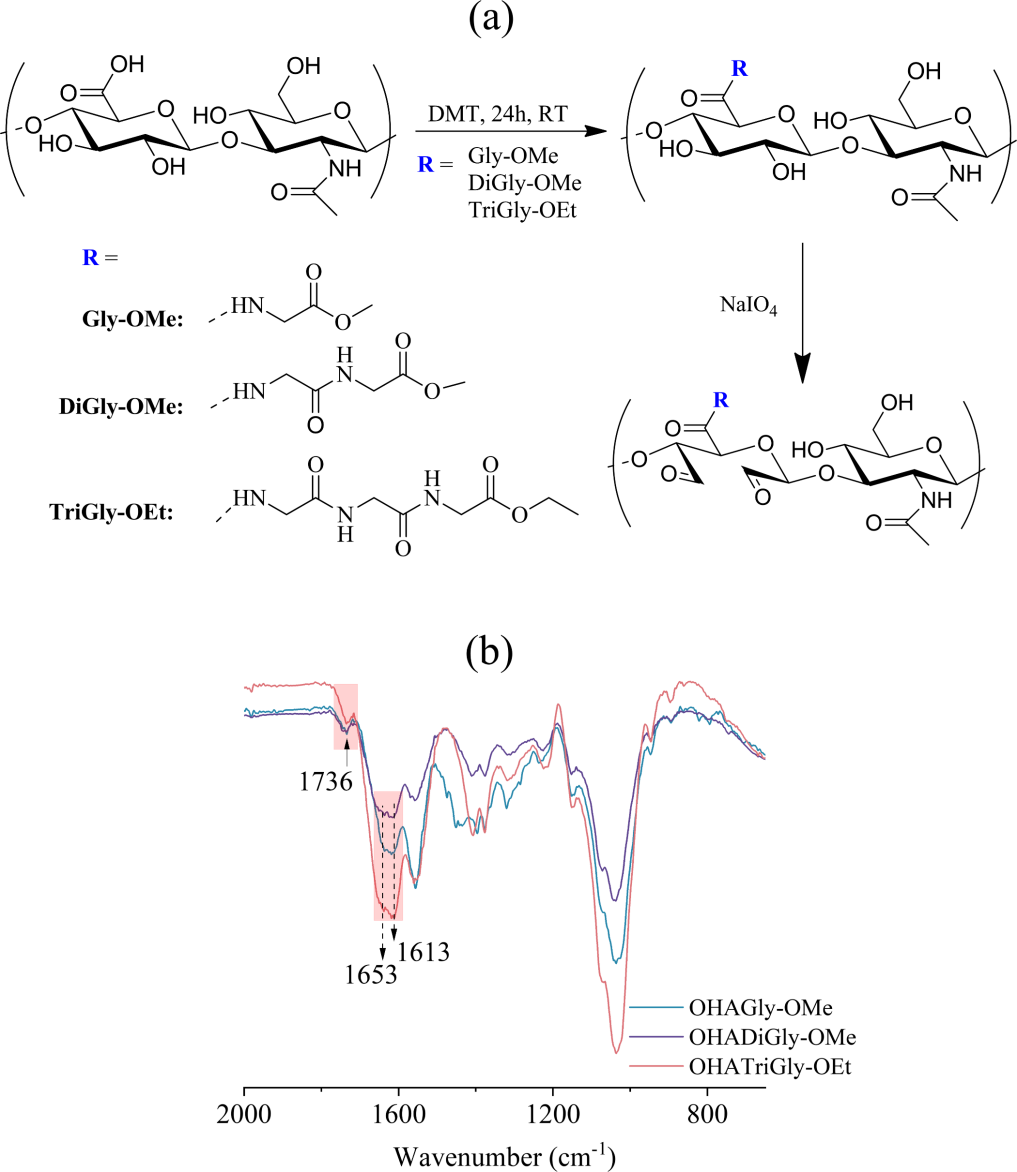
(a) Schematic illustration of synthesis and oxidation of hyaluronic acid glycine/glycine peptide conjugates and (b) infrared spectroscopy of oxidized hyaluronic acid glycine/glycine peptide conjugates.

Figure 1a (top) illustrates the schematic representation of the conjugation of glycine (Gly-OMe), glycine dipeptides (DiGly-OMe) and glycine tripeptides (TriGly-OEt) to HA via amide bonds. The conjugation was performed in an aqueous environment at room temperature using DMT as a coupling agent.^39^ The successful conjugation of glycine/glycine peptides to HA was confirmed by infrared analysis. Characteristics peaks of HA (Figure S1) were identified at 2881 cm^–1^ (C–H stretching) and 1605 cm^–1^ (carboxyl carbonyl group). For glycine and glycine peptides (Figure S2), the carbonyl stretching vibrations of the ester bonds were observed at 1742 cm^–1^ (Gly-OMe), 1692 cm^–1^ (DiGly-OMe), 1642 cm^–1^ (TriGly-OEt). The amide bonds formed between HA and glycine and glycine peptides were identified by the carbonyl peaks between 1660 and 1650 cm^–1^ (amide I) in all conjugates. These signals overlap with the amide bonds of peptide in HAGly-OMe, HADiGly-OMe and HATriGly-OEt (see Figure S1). For more detailed information on the characterization, including NMR, can be found in the literature published elsewhere.^39^

### 3.2 Oxidation of Hyaluronic Acid Glycine/Glycine Peptide Conjugates

To introduce aldehyde functional groups into HA, the oxidation of purified and isolated HA-glycine/glycine peptide conjugates was performed using NaIO_4_, following a previously established protocol.^56,61^ This method involves cleaving the vicinal diols (adjacent hydroxyl groups) within the HA backbone with periodate, leading to the formation of aldehyde groups (see Figure 1a). These aldehyde groups offer versatile sites for subsequent chemical modification.^62^ The presence of aldehyde groups can be confirmed by an infrared peak at 1736 cm^–1^ (Figure 1b).^63−65^ However, this peak also appears in the HA-glycine/glycine peptide conjugates. overlapping with ester peaks of glycine and glycine peptides in the conjugates (see Figure S2) and making interpretation difficult. To address this, we performed a ninhydrin test to quantify the formed aldehyde groups in the conjugates.^55^ The number of aldehyde groups was determined by reacting the conjugates with glycine via Schiff-base chemistry,^62,66^ which involves the formation of a Schiff base adduct between the aldehyde groups and the amino groups of glycine, resulting in a colored compound. The latter can be quantitatively measured to ascertain the concentration of aldehyde groups present in the HA polymer conjugates. Subsequently, the amount of glycine remaining in the solution was analyzed, and a calibration curve was established (see Figure S3). The yield of Schiff-base chemistry was calculated as 61.87%, 59.17%, and 57.42% for OHAGly-OMe, OHADiGly-OMe, and OHATriGly-OEt, respectively. The oxidation degree achieved in this study for the aforementioned conjugates is higher or comparable to the published works in the literature.^63−65^

### 3.3 Functionalization of PCL Scaffolds with OHA-Glycine and Glycine-Peptide Conjugates

#### 3.3.1 Aminolysis of 3D Printed PCL Scaffolds

**Figure 2:**
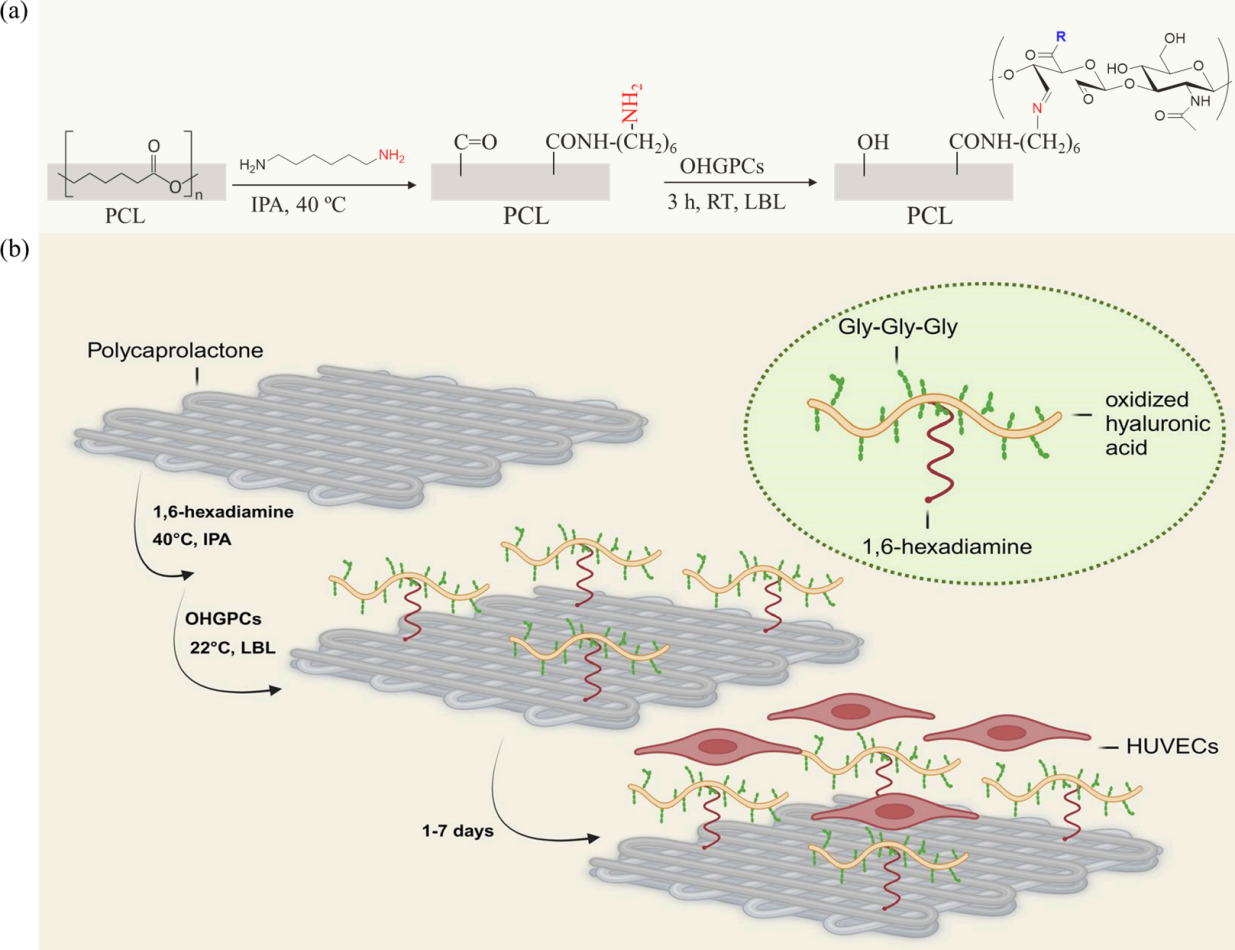
(a) Schematic illustration of conjugation of oxidized hyaluronic acid glycine–peptide conjugates (OHAGPCs) to aminolyzed PCL scaffold and (b) their impact on endothelial cell adhesion.

**Figure 3:**
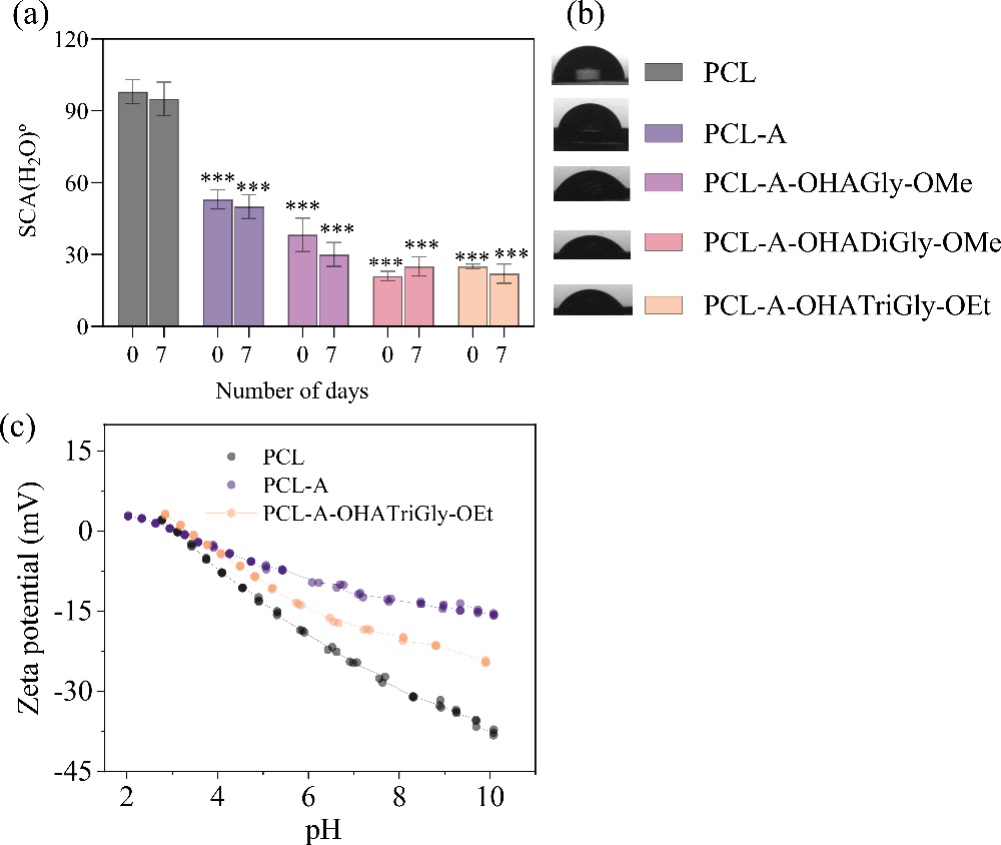
(a–b) Static water contact angle (SCA(H_2_O)) values and (c) zeta-potential of PCL scaffold before and after aminolysis and conjugation with oxidized hyaluronic acid glycine/glycine-peptides; data analysis was done by one-ANOVA with Dunnett test, values are presented as ± SD; ** *p* < 0.05, *** *p* < 0.05 (compared to control PCL).

The PCL scaffolds were aminolyzed using 1,6-hexanediamine to introduce reactive amino groups onto the PCL matrix (Figure 2). In this chemical process, the carbonyl group within the PCL chain was subject to nucleophilic attack by the amine group of the 1,6-hexanediamine compound. Consequently, an amide linkage formed between the PCL chain and the amine compound, facilitating the integration of amino functionality into the PCL backbone.^12,14,21^ The successful aminolysis of PCL was corroborated through static water contact angle measurements (SCA(H_2_O)), which showed a reduction from 98 ± 5° to 63 ± 4° after treatment (Figure 3a,b). This decrease indicates that the originally hydrophobic PCL surface became more hydrophilic. These results align with previously reported data.^12^ Moreover, the amount of amino groups (−NH_2_) introduced in the aminolyzed 3D-printed PCL was quantified via the ninhydrin and XPS measurements, yielding a concentration of 0.158 ± 0.042 M/g of PCL scaffold and 8.1 at. % (Table 1) respectively. Subsequent streaming potential measurements revealed a decrease in zeta-potential from −26.32 mV to −12.04 mV at a pH value of 7.4 (see Figure 3c), indicating that the incorporation of positively charged amino groups partially neutralized the scaffold’s surface charge. These zeta potential changes reflect how surface modification has altered the scaffold’s charge properties, potentially enhancing cell attachment and protein interactions, consistent with previous studies.^54^

**Table 1:** Surface Atomic Concentrations of C, O, N for Untreated, Aminolyzed and OHA-Glycine Peptide Conjugated PCL Scaffolds

	Surface atomic concentration (%)		
Element	PCL	PCL-A	PCL-A-OHAGly-OMe
C	79.0	67.6	69.2
O	21.0	24.3	25.2
N	–	8.1	5.6

#### 3.3.2 Conjugation of Oxidized HA-Glycine/Glycine-Peptides to Aminolyzed PCL Scaffolds

The PCL scaffolds, augmented with amino groups via an aminolysis reaction, serve as the basis for the conjugation process involving oxidized HA-glycine and HA-glycine peptides. This conjugation can be facilitated through Schiff-base chemistry,^56,63,66^ wherein the aldehyde groups of oxidized HA engage in reactions with the amino groups on the PCL surface, forming robust covalent linkages known as Schiff bases. The chemical functionalization of aminolyzed PCL (PCL-A) with these conjugates was confirmed through various analytical methods. For instance, the wettability of PCL-A (SCA(H_2_O): 63 ± 4°) decreased significantly after conjugation with OHA-glycine/glycine peptide conjugates (see Figure 3a,b). Specifically, the OHAGly-OMe conjugated surfaces exhibited a value of 38 ± 7°, while OHADiGly-OMe and OHATriGly-OEt conjugated surfaces showed values of 21 ± 2° and 25 ± 1°, respectively. The slight increase in the SCA(H_2_O)) observed for OHATriGly-OEt may be attributed to reduced surface coverage at the PCL scaffold surface. This decreased surface coverage could result in a more hydrophobic surface, leading to the observed increase in the water contact angle. Overall, these results confirm the successful chemical attachment of all three conjugates to the PCL-A scaffolds via Schiff-base chemistry. Additionally, the stability of these attached conjugates was evaluated by incubating them in cell culture medium (ADMEM) at 37 °C for 7 days under dynamic conditions. The subsequent measurement of SCA(H_2_O) revealed values (OHAGly-OMe: 30 ± 5°; OHADiGly-OMe: 25 ± 4°; OHATriGly-OEt: 22 ± 4°) similar to those obtained prior to the stability test, indicating that all conjugates retained their attachment, demonstrating both the durability and stability of the modified PCL-A surfaces.

**Figure 4:**
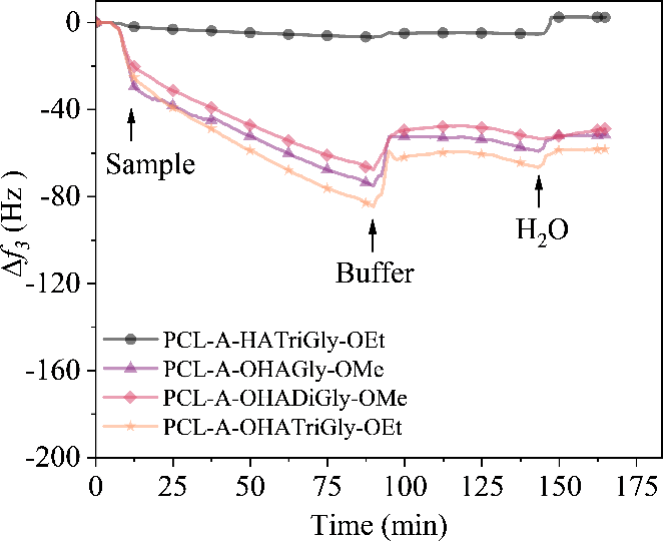
QCM-D frequency change for the conjugation of OHA-glycine/glycine peptide conjugates onto the aminolyzed PCL thin films.

To confirm the covalent attachment of OHA-glycine/glycine peptide conjugates to aminolyzed PCL thin films (PCL-A) via Schiff-base reaction, QCM-D experiments were performed (see Figure 4). This experiment enables real-time monitoring of surface reactions, providing insights into the mass and binding dynamics of the conjugates with the amino groups on PCL-A.^67,68^ As shown in Figure 4, when OHA-glycine/glycine peptide conjugates were introduced to the PCL-A surface, a notable frequency decrease was observed compared to the unmodified PCL, indicating mass (conjugates) attachment. Following rinsing with carbonate buffer and water, the frequency of the neat (unmodified) PCL returned to baseline, while aminolyzed PCL-A showed no substantial frequency recovery, suggesting that the OHA-glycine/glycine peptide conjugates were irreversibly attached to PCL-A’s amino groups via stable imine bonds. To validate the specificity of this attachment, nonoxidized HA-glycine/glycine peptide conjugates were introduced to the PCL-A surface; in this case, no significant frequency decrease was detected, reinforcing that OHA-glycine/glycine peptide conjugates attached to PCL-A covalently through imine bond formation, rather than through noncovalent interactions such as electrostatic forces. Among the conjugates tested, OHA-TriGly-OEt showed a mass increase of approximately 10.3 mg m^–2^ (−58 Hz), while OHA-Gly-OMe and OHA-DiGly-OMe exhibited mass increases in the range of 8.5–9.1 mg m^–2^ (approximately −48 to −51 Hz). This study highlights the utility of aminolysis to introduce amino groups on the PCL surface, enabling Schiff-base linkage of OHA-glycine/glycine peptide conjugates. This covalent attachment approach provides a stable, biomimetic microenvironment that closely resembles the native extracellular matrix (ECM), promoting enhanced endothelial cell (EC) adhesion and growth.

**Figure 5:**
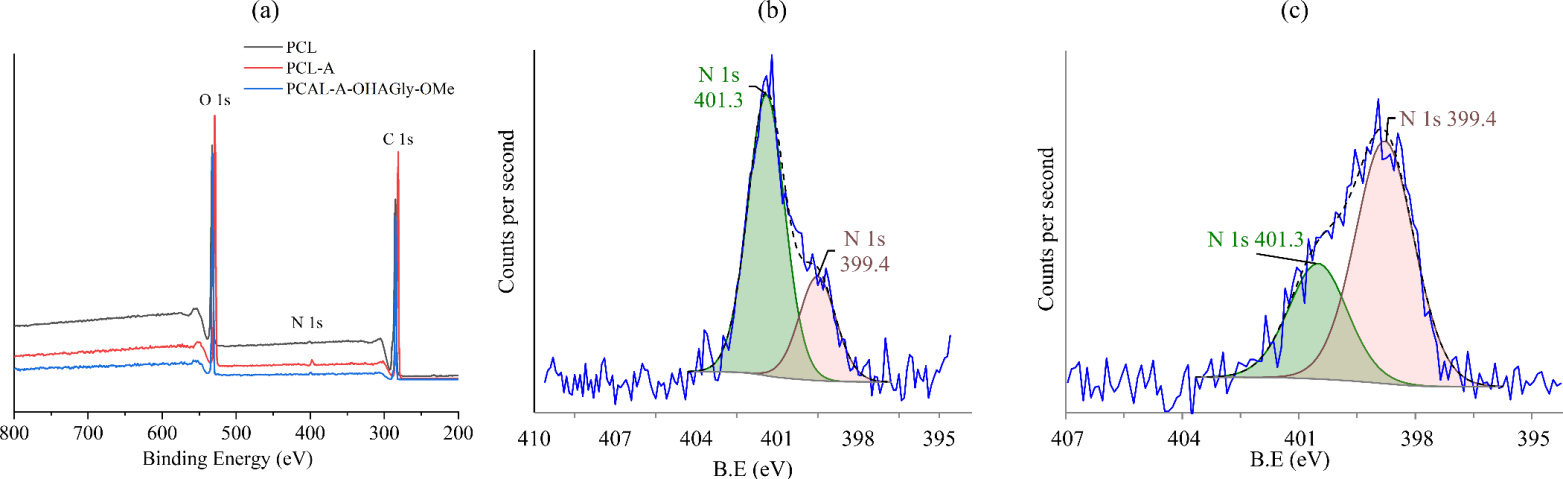
(a) Survey spectra of PCL, PCL-A and OHAGly-OMe conjugated PCL-A scaffolds, (b) fitted N 1s spectra of PCL-A, and (c) fitted N 1s spectra of OHAGly-OMe conjugated PCL-A scaffold before sputtering; the blue lines in b and c represent the measured high-resolution spectra, and the dashed lines determine the Shirley background.

The aminolysis and conjugation of OHAGly conjugates to PCL were further confirmed using XPS spectroscopy. The survey spectrum in Figure 5a shows C 1s (surface atomic concentration of 79.0 at. %, Table 1) and O 1s (21.0 at. %) peaks for the untreated PCL. Compared to untreated PCL, the survey spectrum for PCL-A shows the emergence of a N 1s peak (8.1 at. %) alongside the C 1s and O 1s peaks. This indicates the successful introduction of amino groups onto the PCL scaffolds via aminolysis. The presence of the N 1s peak was also found for the OHAGly-OMe conjugated scaffold, however the surface atomic concentration of N for the latter was approximately 2.5 at. % lower compared to PCL-A. This difference can be attributed to the surface-sensitive nature of XPS (the topmost position contains a lower amount than the bulk), the relatively lower nitrogen content in HA, and possible interactions and coverage variations of the HA coating.^69^ For clarity, only the OHAGly-OMe conjugated samples were chosen for the XPS analysis.

Figure S4 shows the XPS depth profile of PCL-A and OHAGly-OMe-conjugated PCL-A scaffolds. For the PCL-A sample, the N 1s peak can be deconvoluted into two peaks at binding energies of 399.4 and 401.3 eV (Figure 5b), corresponding to the O=C—NH (amide) and C-NH_2_ (primary amine) groups, respectively. Before sputtering, the peak for O=C—NH was more intense than the peak for C-NH_2_, suggesting that more amide groups were on the surface than primary amine groups. On the other hand, the N 1s spectrum for OHAGly-OMe-conjugated scaffold can also be fitted with the peaks at binding energies of 399.4 and 401.3 eV, whereas the peak at 399.4 eV (O=C—NH, amide) was more intense, likely due to the more amide compared to primary amine groups in HA/OHAGly-OMe conjugate (Figure 5c).

**Figure 6:**
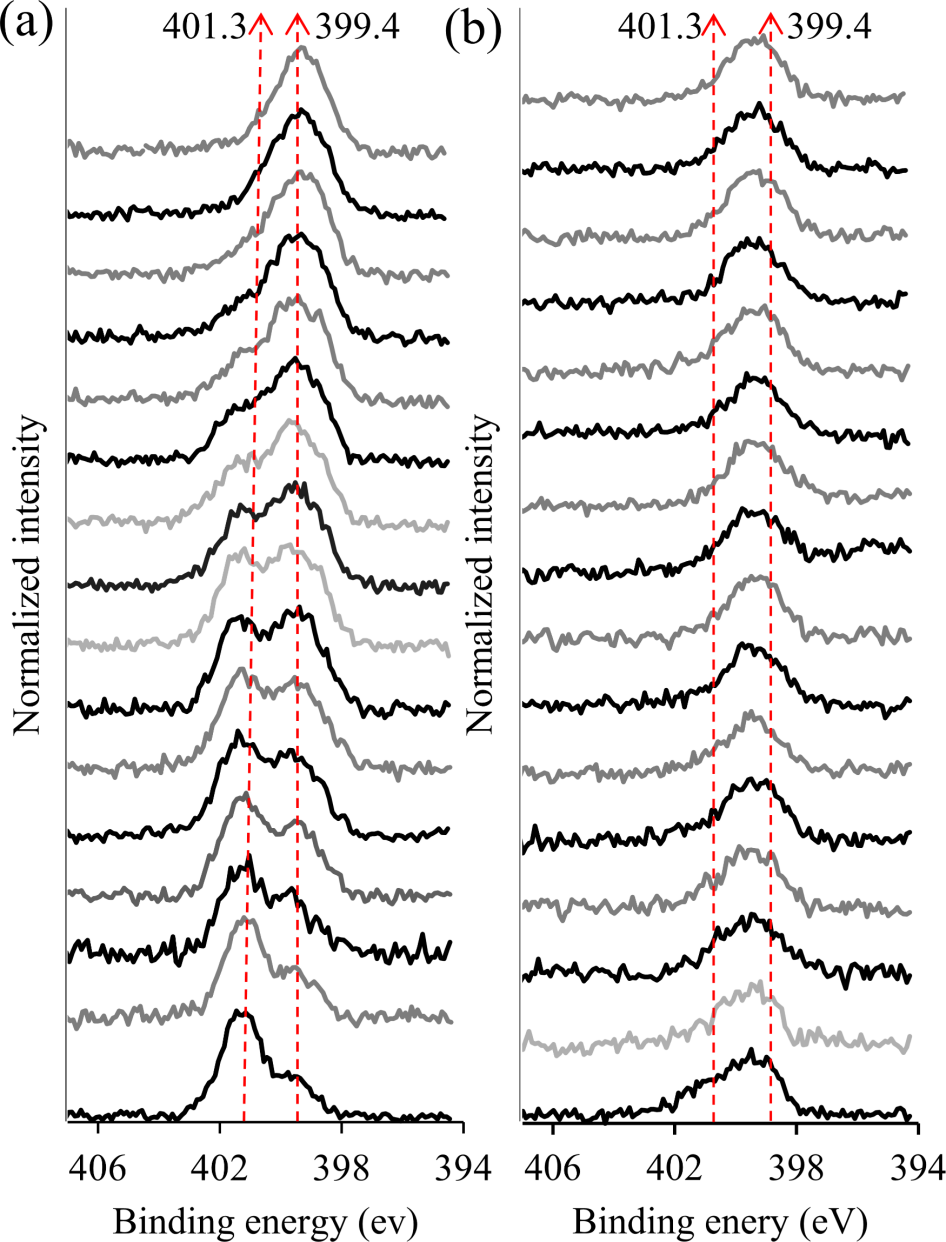
High-resolution N 1s XPS spectra for PCL-A (a) and PCL-A-OHAGly (b) measured during the sputtering with Ar_20000_^+^. The bottom spectra represent the measurement before sputtering.

To analyze the in-depth elemental composition of OHAGly-OMe to PCL-A, high-resolution N 1s spectra were measured (Figure 6) during sputtering with 10 keV Ar_2000_^+^. The N 1s spectrum measured before sputtering comprised two spectral features as mentioned above: O=C—NH (at 399.4 eV) and C-NH_2_ (at 401.3 eV) for the PCL-A sample (the lowest spectrum in Figure 6a). As sputtering progressed, the intensity of the peak at 401.3 eV decreased while the intensity of the peak at 399.4 eV increased, indicating that the top layers of the scaffolds predominantly contain a mixture of both primary amine (C–NH_2_) and amide (O=C—NH) functional groups. In the underlying layers, the intensity of amide functional groups became more pronounced than the primary amine. Only the presence of amide bonds was observed in the deeper layers at the end of sputtering. On the contrary, in the case of OHAGly-OMe conjugated scaffold (Figure 6b), before sputtering, the peak at 401.3 eV was not intense, while the peak at 399.4 eV became more predominant in the underlying layer. It is our opinion that the peak at 399.4 eV is related to the imine bond (C=N) formed between the primary amine groups on PCL-A and the aldehyde groups of OHAGly-OMe conjugate and not due to the amide bonds in HA. The imine bond has a binding energy close to other nitrogen and carbon-containing bonds, such as amines (C–NH_2_) and amides (O=C—NH), leading to peak overlap and making it challenging to distinguish the imine bond from these other species. This overlap has been observed in other studies, such as the reaction of poly(allylamine) with trifluoroacetone,^70^ reaction of 1,5-diaminonaphthalene with 2,4,6-tris(4-formylphenoxy)-1,3,5-triazine^71^ and the hydrogenated carbon nitride film deposition by N_2_/CH^4^ plasma.^72^

#### 3.3.3 Swelling and Degradation Studies

**Figure 7:**
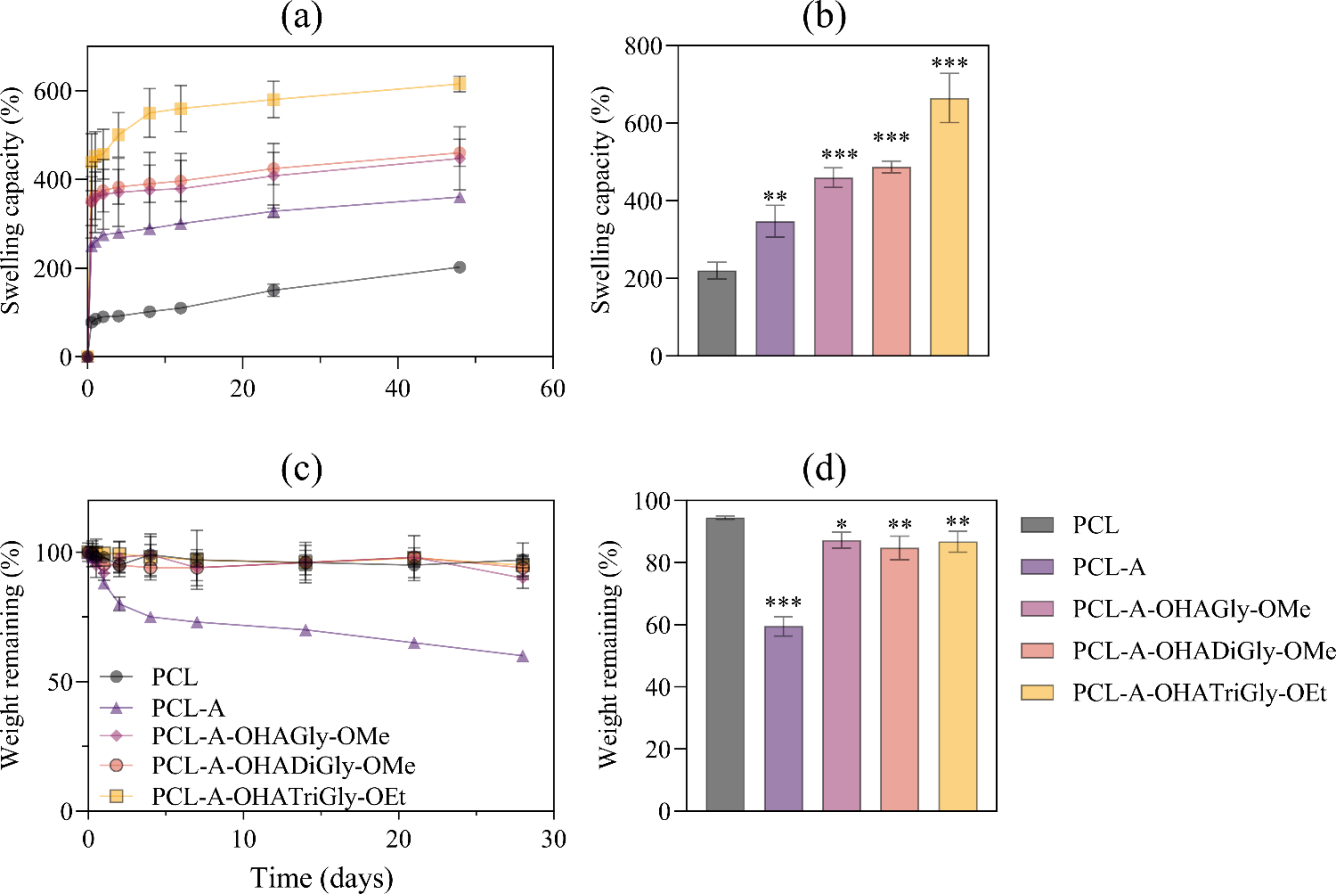
Swelling (a, b) and degradation (c, d) capacity of neat, aminolyzed and OHA-glycine/glycine peptide conjugated scaffolds; data analysis was done by one-ANOVA with Dunnett test, values are presented as ± SD; ** *p* < 0.05, *** *p* < 0.05 (compared to control PCL).

The swelling capacity of polymeric scaffolds is a critical factor for their suitability in TE applications, as it provides an aqueous environment that supports nutrient and metabolite transport essential for cell growth.^73^ This study evaluated the swelling behavior of neat PCL, PCL-A, and OHA-glycine/glycine peptide-conjugated scaffolds in a cell growth medium at 37 °C. As shown in Figure 7a, the uptake of biofluids by the scaffolds increased rapidly during the first hour, then gradually slowed. Neat PCL reached a steady-state after 3 h, while the OHA-glycine/glycine peptide-conjugated scaffolds and PCL-A did not reach a steady state even after 48 h. Notably, all OHA-glycine/glycine peptide-conjugated scaffolds displayed a significantly higher swelling capacity than neat PCL and PCL-A scaffolds. This enhanced swelling capacity is attributed to the presence of hydrophilic functional groups - such as hydroxyl, carboxyl, amine, and amide-in the OHA-glycine/glycine peptide conjugates, as well as the amine groups in PCL-A, which allow greater water molecule binding.^74,75^ Additionally, scaffolds conjugated with a higher number of glycine units demonstrated increased swelling. Overall, the OHA-glycine/glycine peptide-conjugated scaffolds exhibited a 53% increase in swelling capacity compared to neat PCL and PCL-A scaffolds. The swelling capacities of the bioscaffolds are ranked as follows (see Figure 7b): PCL (523 ± 71 g/g) > PCL-A (460 ± 30 g/g) > PCL-A-OHA-Gly-OMe (447 ± 16 g/g) > PCL-A-OHA-DiGly-OMe (343 ± 15 g/g) > PCL-A-OHA-TriGly-OEt. In comparison with literature data, this study’s findings align with existing knowledge that incorporating hydrophilic groups into polymeric scaffolds enhances their swelling properties, which is crucial for optimizing conditions for cell growth and nutrient exchange in TE applications. In a study by Binaymotlagh et al., hydrophilic carboxyl and amine groups in peptide-conjugated scaffolds were found to significantly improve water uptake, with a reported increase in swelling capacity by 45% compared to nonconjugated scaffolds.^76^ Studies like those by Feng et al., have similarly reported that hydrophilic peptide modifications extend the swelling equilibrium time due to the additional hydrogen bonding sites.^77^ Our study reported that the swelling capacity increases with the number of glycine units corroborates literature emphasizing the role of peptide chain length. For example, Cheng et al., showed that scaffolds conjugated with longer peptide chains exhibited improved water retention due to higher hydrophilicity and enhanced polymer–water interactions.^78^

The results of the in vitro degradation study for all scaffolds in biofluid at 37 °C over various time periods are shown in Figures 7c and 7d. Notably, none of the scaffolds were fully degraded after 28 days. Except for the aminolyzed PCL (PCL-A) scaffold, all other scaffolds exhibited minimal degradation, with mass losses of less than 10% after 28 days (Figure 7d). The neat PCL (control) scaffold demonstrated remarkable stability within the cell culture environment. In contrast, the PCL-A scaffold showed reduced stability, with a mass loss of approximately 40% after 28 days. The increased degradation of PCL-A is likely due to the aminolysis process, which introduces amino groups by breaking some ester bonds,^20^ slightly disrupting the original polymer structure. This microstructural alteration can increase the scaffold’s susceptibility to hydrolytic degradation by facilitating excessive water absorption. Furthermore, cell culture media typically contain enzymes secreted by cells (such as lipases and esterases) that accelerate the degradation of ester linkages in PCL.^79^ The aminolyzed surface may interact more readily with these enzymes, enhancing their activity and further increasing degradation rates. Interestingly, scaffolds functionalized with OHA-glycine/glycine peptide conjugates showed no significant weight loss, maintaining stability similar to that of neat PCL. This stability in the aminolyzed PCL after functionalization is likely due to the addition of functional groups that preserve or slightly increase the overall mass without inducing significant degradation. The functionalization likely alters only the surface chemistry while preserving the bulk structure, thereby maintaining the scaffold’s weight.

In conclusion, the bioscaffolds produced in this study, particularly those functionalized with OHA-peptide conjugates, show strong potential for long-term cell growth experiments due to their stability in biofluids.

While the neat PCL exhibits slow degradation due to its hydrophobic nature and crystalline structure, which hinder hydrolytic chain scission in aqueous environments,^80^ the amine groups in PCL-A, disrupt the polymer’s crystalline domains, increasing susceptibility to hydrolytic degradation according to Kweon et al.^81^ This process also enhances water uptake, accelerating degradation. Functionalization of PCL with peptides or other biomolecules often improves surface properties without significantly affecting bulk degradation. Studies like those by Stella et al. highlights how peptide modifications can enhance cellular property while preserving scaffold integrity.^82^ In summary, the experimental findings align with previous studies, confirming that aminolyzed PCL scaffolds degrade more rapidly due to structural changes and enzyme interactions, whereas neat PCL maintains stability. Functionalization with certain molecules can protect the scaffold by altering its surface properties, reducing degradation rates.

#### 3.3.4 Mechanical Properties

**Figure 8:**
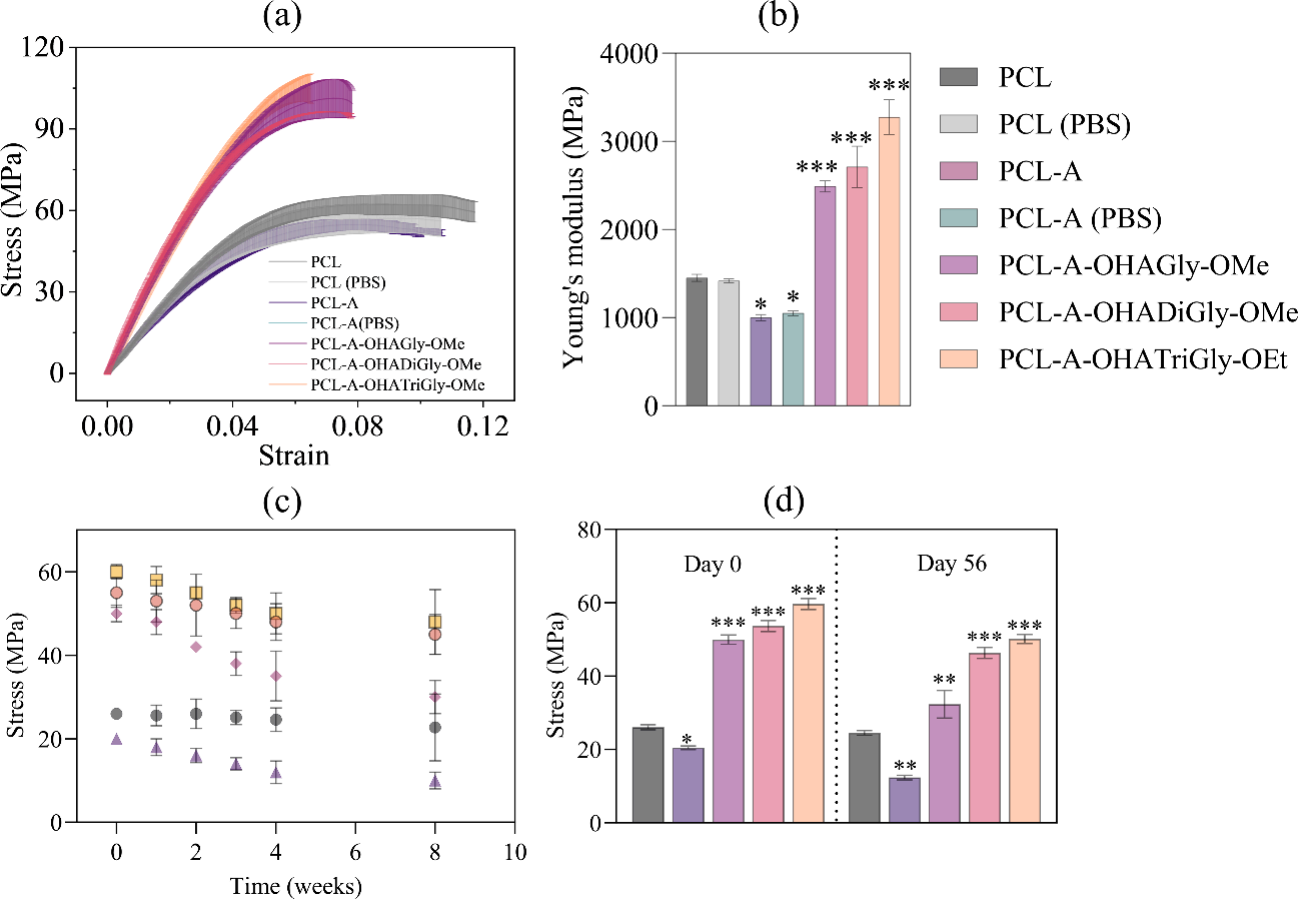
Mechanical properties of neat PCL and functionalized PCL-A scaffolds. (a) Stress–Strain curves and (b) Young’s modulus of neat PCL and PCL-A scaffold conjugated with OHA-glycine/glycine peptide conjugates in dry and wet state (ADMEM). (c, d) Tensile stress/strength evaluation of OHA-glycine/glycine peptide conjugated PCL-A scaffolds evolution during degradation; data analysis was done by one-ANOVA with Dunnett test, values are presented as ± SD; ** *p* < 0.05, *** *p* < 0.05 (compared to control PCL).

While the surface properties are critical for small-diameter cardiovascular applications, as they directly influence endothelial cell interactions, thrombogenicity, and overall cellular adaptation. However, bulk mechanical properties are equally relevant, as they determine the material’s ability to withstand physiological forces, such as blood pressure, without failing or deforming excessively. Optimal (bulk)mechanical characteristics ensure the scaffolds can provide adequate support for cell or tissue growth, mimic the native tissue’s mechanical environment, and withstand physiological stresses. Therefore, in this work, we assessed the tensile strength of 3D printed PCL scaffolds before and after functionalization with OHA-glycine/glycine peptide conjugates. For this experiment, the samples were immersed in PBS buffer for 7 days at pH 7.4 at 37 °C. The widespread use of PCL is hindered by its extremely low Young’s modulus (stiffness), as it is known to exhibit ductile behavior.^83^Figure 8a shows the stress–strain curves of the PCL-A attached with OHA-glycine/glycine peptide conjugates. The untreated (dry) PCL scaffold exhibited a tensile strength of 25.9 ± 3.2 MPa, which was consistent for PCL scaffolds immersed in PBS buffer (Figure 8a). However, the scaffold PCL-A showed a slight reduction in tensile strength, likely due to the chain scission, disruption of crystalline regions, chemical degradation, and potential phase separation introduced during the aminolysis process. The aminolysis process may introduce structural defects or irregularities on the polymer surface, which can act as points of weakness when the material is subjected to tensile forces.^84^ These defects make the polymer more prone to failure under mechanical stress. This observation aligns with other studies on the aminolysis of PCL electrospun nanofibers^12^ and films.^85^ Interestingly, the OHAGly-OMe functionalized scaffolds showed a 2-fold increased tensile strength (50.7 ± 4.1 MPa) compared to both untreated PCL or PCL-A, which is consistent with previous studies where chemically modified PCL exhibited similar improvements. For example, PCL scaffolds functionalized with gelatin demonstrated a significant increase in tensile strength from 25 to 50 MPa, as reported by Zheng et al.^86^ Similarly, PCL grafted with collagen, as noted by Li et al., also showed improved mechanical properties and enhanced degradation resistance,^87^ demonstrating the advantages of chemical modifications in improving PCL’s structural integrity. The increase in tensile strength was more pronounced with a higher number of glycine molecules in the conjugates. It is suggested that the amine groups on PCL in an aqueous environment like PBS can react with aldehyde groups of OHA-glycine peptide conjugates to form Schiff bases (imines) or amide bonds, as evidenced by the XPS results. This reaction results in a cross-linked network that enhances the mechanical properties by increasing the material’s structural integrity and resistance to deformation.^88^ Grafting PCL with OHA-glycine peptides significantly enhanced the interfacial adhesion between the PCL matrix and the peptides. As a result, the mechanical properties were notably improved, establishing OHA-glycine peptides as an excellent effective adhesive coating. Additionally, the Young’s modulus increased for the functionalized scaffold compared to the untreated PCL (Figure 8b). This can be attributed to the cross-linking, which increases the rigidity and mechanical strength of the polymer network, thus contributing to a higher Young’s modulus. Overall, the Young’s modulus and ultimate tensile strength increased with the number of glycine units in the conjugate, indicating that the mechanical behavior of the scaffold is closely linked to glycine content. Scaffolds grafted with the OHATriGly-OEt conjugate exhibited the highest stiffness and mechanical properties, characterized by the highest Young’s modulus. The effect of surface grafting of the conjugates enhances the bulk tensile properties of PCL scaffold by improving interfacial adhesion, promoting cross-linking near the surface, regulating swelling, enhancing stress transfer, and modifying the polymer’s crystalline structure.^89^ These effects result in better load distribution, reduced defect propagation, and increased strength and stiffness.^90^ Consequently, the prepared materials demonstrated commendable stiffness and yet it remains within an appropriate range to maintain compliance suitable for small-diameter vascular grafts. Similar results in tensile strength and Young’s modulus have been reported by other authors. For example, PCL scaffolds functionalized with gelatin, collagen or cellulose via chemical routes have shown significant improvements in mechanical properties compared to untreated ones.^91,92^ For cardiovascular applications, mechanical properties must balance flexibility and strength to ensure compliance similar to native vessels while avoiding excessive stiffness, which could lead to mismatch and complications like intimal hyperplasia. While the coating slightly increases the material’s stiffness, it remains within an appropriate range to maintain compliance suitable for small-diameter vascular grafts.

The time evolution of tensile strength is shown in Figures 8c and 8d. According to these figures, the tensile strength of neat PCL remained constant at approximately 25 MPa throughout the 8-week period. In contrast, the tensile strength of PCL-A decreased by 40%, reaching 15 MPa after 8 weeks. Notably, the conjugated PCL-A scaffolds exhibited significantly higher tensile strength, reaching a maximum value of 54 MPa at the end of 8 weeks. The mechanical stability of these scaffolds improved with increasing amounts of glycine in the conjugated scaffolds. These results clearly indicate that OHA-glycine/glycine peptide conjugation to PCL-A provided enhanced structural support and resilience, helping to maintain the mechanical integrity of the PCL scaffold during degradation, thereby reducing the loss in mechanical strength. Moreover, the improved mechanical stability during degradation, as observed with OHA-glycine conjugated scaffolds, is consistent with findings in the literature, such as those by Zhao et al., who reported that the functionalization of PCL with polysaccharides significantly reduced the loss of tensile strength over time.^93^ This is especially important for biomedical applications where long-term mechanical integrity is crucial, such as in vascular grafts. For instance, PCL scaffolds functionalized with cellulose were shown to maintain mechanical strength even after prolonged degradation, which aligns with the observed resilience of our OHA-glycine conjugated PCL scaffolds.

In conclusion, the integration of OHA-glycine peptide conjugates with PCL scaffolds offers significant mechanical and structural benefits, which are consistent with similar findings in the literature. These advancements position the functionalized scaffolds as promising candidates for a range of biomedical applications, including tissue engineering and vascular grafts, where both mechanical strength and degradation resistance are critical.

### 3.4 In Vitro Analysis on HUVEC Cells

**Figure 9:**
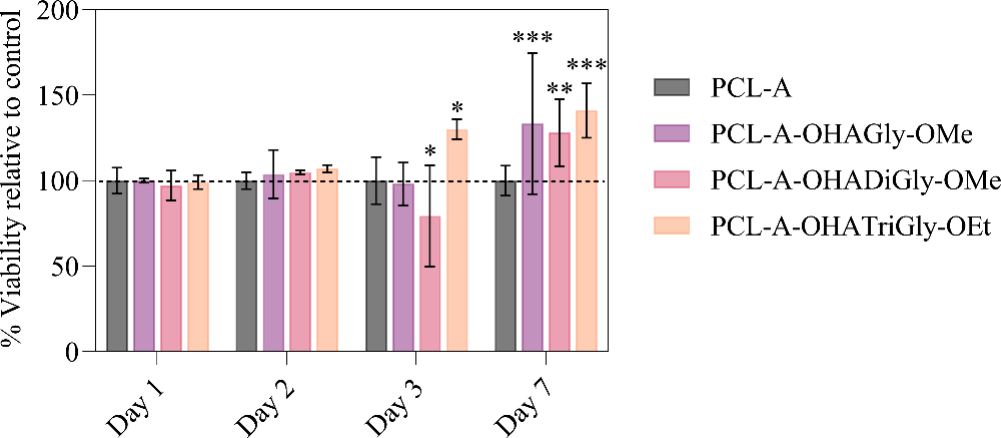
Viability of HUVEC cells cultured on OHA-glycine peptide conjugated PCL scaffolds at different time points. The dotted black line represents the control PCL scaffold; data analysis was done by one-ANOVA with Dunnett test, values are presented as ± SD; *p < 0.05, ** *p* < 0.05, *** *p* < 0.05 (compared to control PCL).

The HUVEC proliferative capacity of PCL scaffolds, modified with OHA glycine/glycine peptide conjugates, were evaluated using HUVEC cells. Figure 9 presents the MTS assay results for HUVEC cells exposed to untreated PCL (control) and OHA-glycine peptide conjugates at different time points. The data indicates that the scaffolds modified with OHA-glycine peptide conjugates did not reduce cell viability compared to the control sample. Moreover, no significant differences in cell viability were observed on days 1 and 2 across all samples relative to the PCL control. However, from days 3 to 7, all conjugates or coatings demonstrated significantly higher cell viability compared to the control and PCL-A samples. These findings highlight the potential of all tested coatings (OHA-Gly-OMe, OHA-DiGly-OMe, and OHA-TriGly-OEt) as biocompatible materials, particularly well-suited for longer-term cell culturing. This emphasizes the biological relevance of surface modifications in enhancing cell viability over extended periods. Overall, the MTS assay demonstrated that the OHA-glycine peptide-conjugated scaffolds are biocompatible and can support cell adhesion and growth over an extended period.

**Figure 10:**
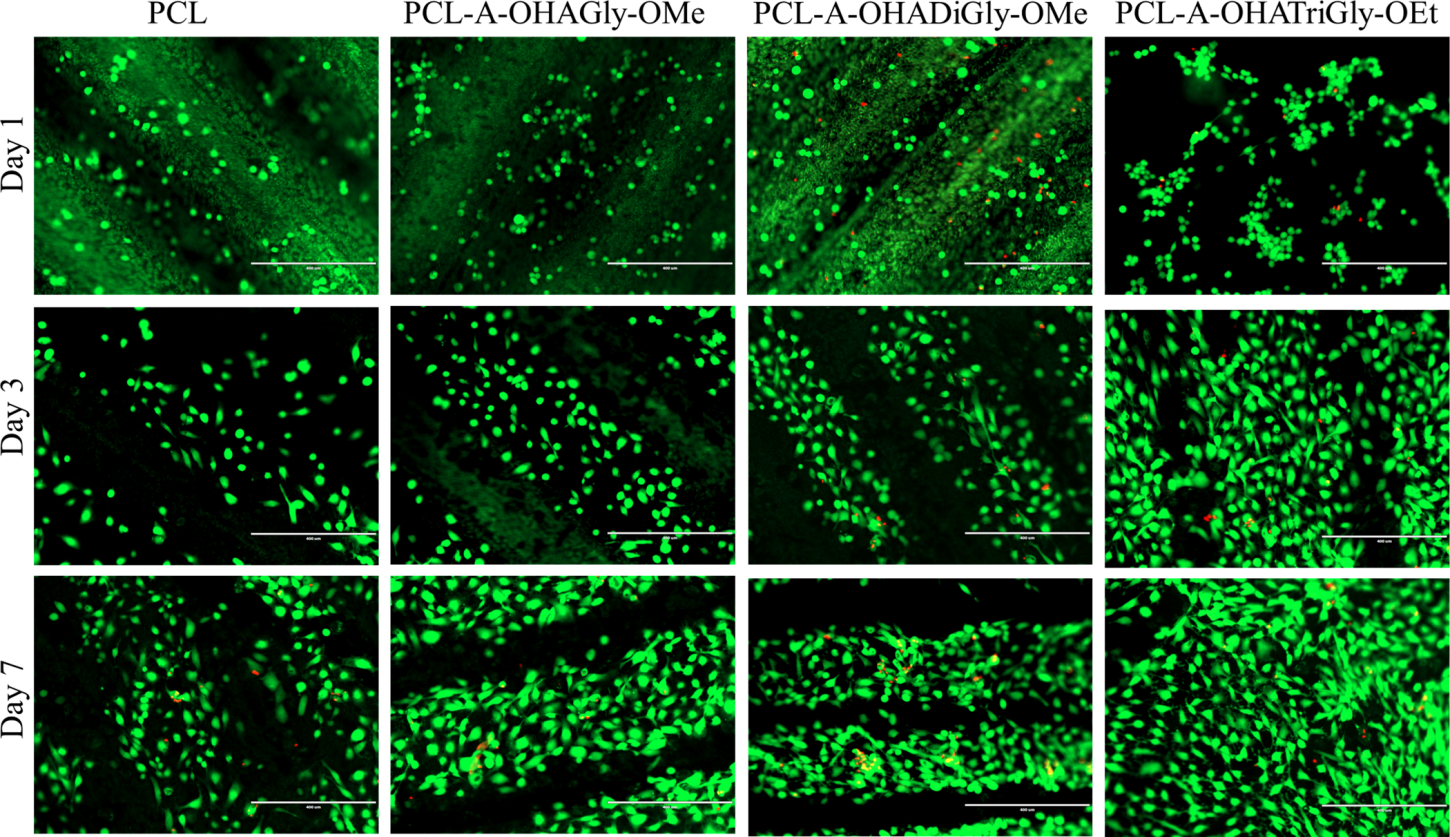
Representative live/dead fluorescence stained HUVECs on OHA-glycine peptide conjugated PCL scaffolds after 1-, 2-, 3- and 7-days culture, scale bar 400 μm.

**Figure 11:**
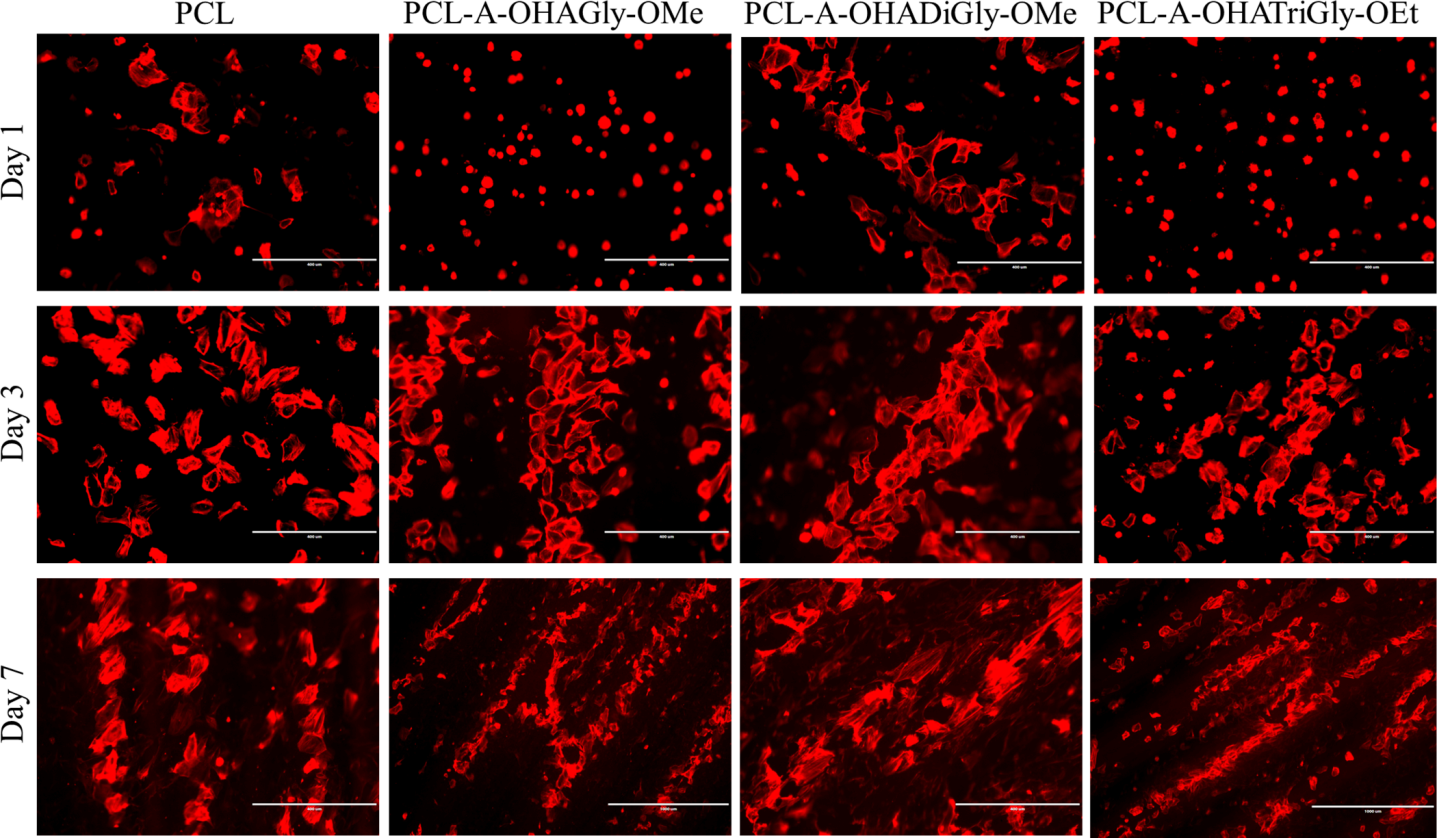
Representative phalloidin stained HUVECs on OHA-glycine peptide conjugated PCL scaffolds after 1-, 2-, 3- and 7-days culture, scale bar 400 μm.

In addition to MTS assay, we also used a live/dead and phalloidin staining assay to validate the cellular response for its adherence and morphology on the scaffold surfaces. The scaffolds were examined using the live/lead assay, which “visually” displays live or dead cells on the scaffold’s surface for 1–7 days (see Figure 10). Although a few dead cells were observed on all scaffolds, the number of viable cells on the scaffold surface increased with respect to culture time. Compared to control sample, more viable cells were observed on the OHA-glycine peptide functionalized scaffolds. In addition, the cell coverage was improved with increasing number of glycine molecules in the OHA-glycine peptide conjugates. This was more pronounced at 7 days. The same was observed with phalloidine staining (Figure 11). On day 7, the cells were better distributed on the OHA-glycine peptide functionalized surfaces compared to untreated PCL (control) sample. Overall, the results showed that all tested scaffolds were nontoxic to cells and allow cell adhesion and proliferation. Our work emphasizes the role of integrin-mediated signaling pathways in EC adhesion, a critical aspect in vascular applications. By selecting glycine, abundant in cell adhesion motifs, we ensure the scaffold’s ability to support cellular attachment and proliferation without the high cost of synthetic ECM peptides.

## 4 Conclusions

In this study, we demonstrated a method for functionalizing 3D-printed PCL scaffolds with HA-glycine/glycine peptide conjugates and explored their potential application in promoting endothelial cell adhesion and growth. The conjugates were oxidized using sodium periodate and subsequently immobilized onto amino groups on the PCL, introduced via an aminolysis reaction. The formation of aldehyde groups in the HA conjugate was evidenced by a peak at 1736 cm^–1^ in the IR spectrum. Ninhydrin assay results indicated degrees of oxidation of 61.87%, 59.17%, and 57.42% for OHAGly-OMe, OHADiGly-OMe, and OHATriGly-OEt, respectively. The water contact angle of PCL was reduced from ca. 98° to 63° after aminolysis, and further to ca. 21° after functionalization with the conjugates. Zeta potential measurements corroborated these findings, showing an increase from −26.32 mV to −12.04 mV postaminolysis and a subsequent decrease to −19.2 mV after functionalization. XPS analysis revealed the presence of both primary amine (C-NH_2_) at 401.3 eV and amide (O=C—NH) peaks at 399.4 eV on the PCL scaffold due to aminolysis, with the amide peak being more pronounced in the underlying layers. While the attachment of the conjugate via an imine bond was indicated by QCM-D, XPS analysis faced challenges due to the energy level overlap of the imine bond with both primary amine and amide bonds. The functionalized scaffolds showed significantly increased swelling compared to untreated PCL, whereas there were no notable differences in weight loss between untreated and functionalized PCL scaffolds. Mechanical testing revealed that PCL (wet) scaffolds functionalized with the conjugates showed significantly enhanced tensile strength and Young’s modulus compared to both untreated and aminolyzed PCL scaffolds. The mechanical properties increased proportionally with the number of glycine units in the conjugate, suggesting a strong correlation between mechanical performance and glycine content. In-vitro cell testing with human umbilical vein endothelial cells (HUVEC) over a period of 1–7 days revealed no toxicity across all tested scaffolds, with increased cell proliferation observed by day 7. Live/dead and phalloidin staining assays confirmed higher cell viability and improved cell coverage on the OHA-glycine peptide functionalized scaffolds, with better performance noted as the number of glycine molecules in the conjugates increased. Overall, the functionalized PCL scaffolds presented in this study demonstrate broad potential in tissue engineering and regenerative medicine. By enhancing endothelial cell activity, mechanical properties, and bioactivity, these scaffolds hold promise for applications such as vascularized tissue constructs, bone and cartilage repair, wound healing, drug delivery systems, organ regeneration, and soft tissue engineering. These advancements establish a versatile platform for improving tissue integration, regeneration, and the development of personalized medical therapies.

## Supporting Information Available

Infrared spectrum of glycine/glycine peptides conjugated to hyaluronic acid, calibration curve of ninhydrin assay and XPS depth profile of aminolyzed PCL before and after conjugation with OHAGly-OMe (PDF)
