# Supplementary material for: Functionalization of Polycaprolactone 3D Scaffolds with Hyaluronic Acid Glycine-Peptide Conjugates and Endothelial Cell Adhesion
Source: Biomacromolecules. 2025 Feb 24;26(3):1771–87. doi: 10.1021/acs.biomac.4c01559 (PMC11898084; doi:10.1021/acs.biomac.4c01559)
Supplement: Supplementary file 1 — bm4c01559_si_001.pdf [file bm4c01559_si_001.pdf]

Supporting Information

Functionalization of Polycaprolactone 3D Scaffolds with Hyaluronic Acid Glycine-Peptide Conjugates and Endothelial Cell Adhesion

Tamilselvan Mohan[†,‡,1,*], Fazilet Gürer[‡,1], Doris Bračič[‡,1], Florian Lackner[†], Chandran Nagaraj[¥], Uroš Maver[β,1], Lidija Gradišnik[β], Matjaž Finšgar[€], Rupert Kargl[†,‡], Karin Stana Kleinschek[†,θ,1,*]

[†]Graz University of Technology, Institute of Chemistry and Technology of Biobased System, Stremayrgasse 9, 8010 Graz, Austria

[‡]University of Maribor, Faculty of Mechanical Engineering, Laboratory for Characterisation and Processing of Polymers, Smetanova ulica17, 2000 Maribor, Slovenia

[β]University of Maribor, Faculty of Medicine, Institute of Biomedical Sciences, Taborska Ulica 8, 2000 Maribor, Slovenia

[¥]Department of Internal Medicine, Division of Pulmonology, Medical University of Graz, 8010 Graz, Austria

[€]University of Maribor, Faculty of Chemistry and Chemical Engineering, Laboratory for Analytical Chemistry and Industrial Analysis, Smetanova ulica 17, 2000 Maribor, Slovenia

[θ]University of Maribor, Institute of Automation, Faculty of Electrical Engineering and Computer Science, , Koroska cesta 46, 2000 Maribor, Slovenia

[1]Members of the European Polysaccharide Network of Excellence (EPNOE), Celestijnenlaan 200 F, 3001 Leuven, Belgium

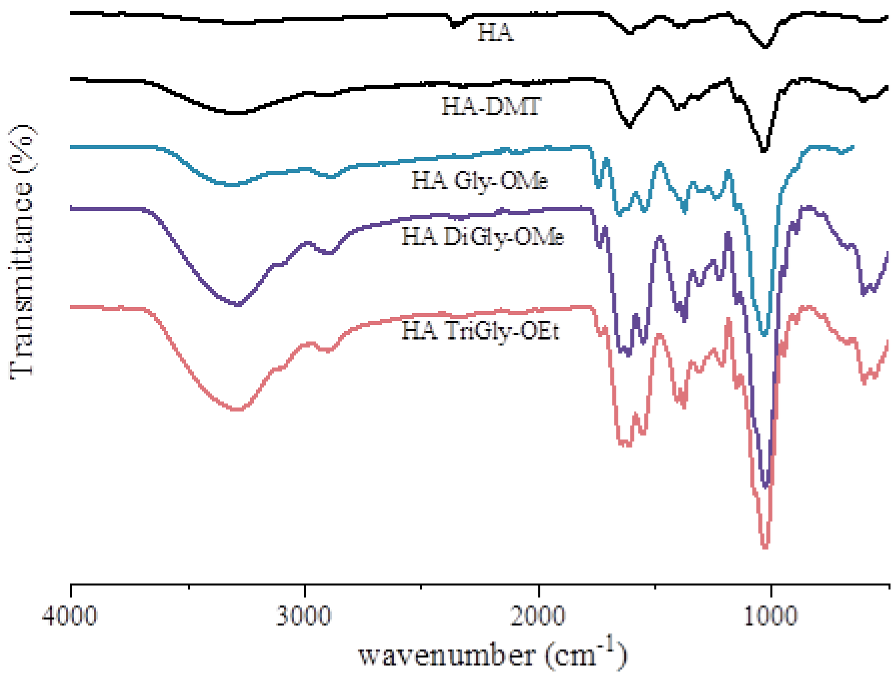
HA
HA-DMT
HA Gly-OMe
HA DiGly-OMe
HA TriGly-OEt
Transmittance (%)
4000
3000
2000
1000
wavenumber (cm-1)

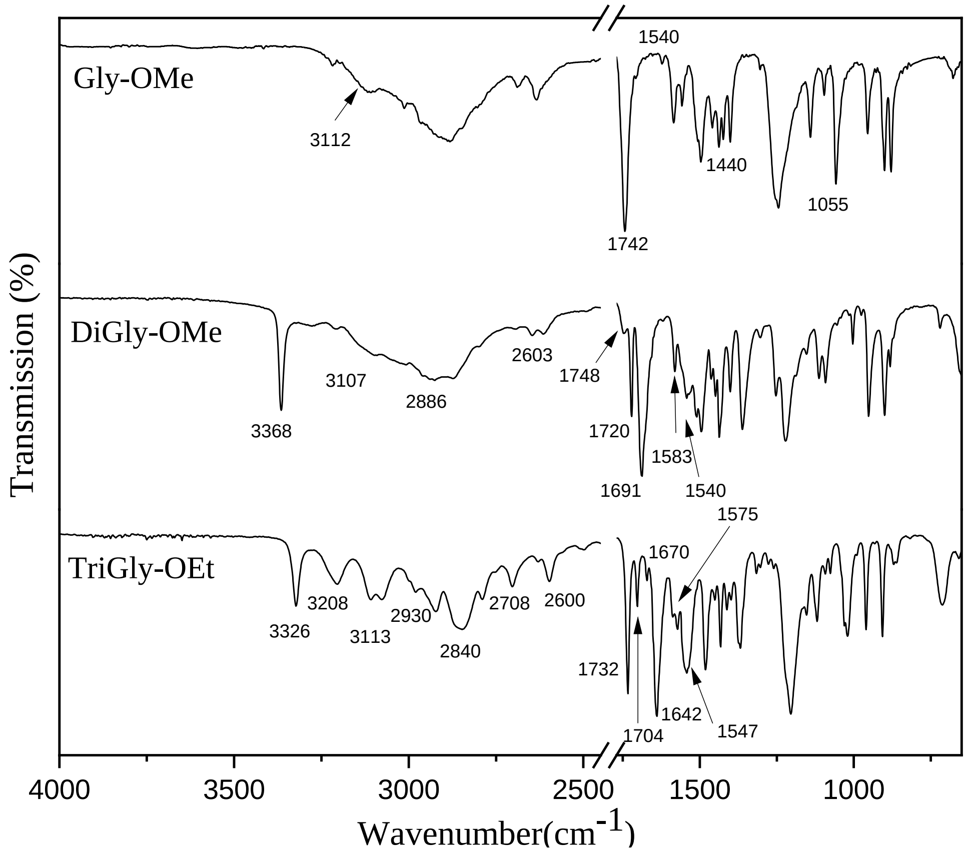
Gly-OMe
3112
1540
1742
1440
1055
DiGly-OMe
3368
3107
2886
2603
1748
1720
1691
1583
1540
TriGly-OEt
3326
3208
3113
2930
2840
2708
2600
1732
1670
1575
1704
1642
1547
Transmission (%)
4000
3500
3000
2500
1500
1000
Wavenumber(cm-1)

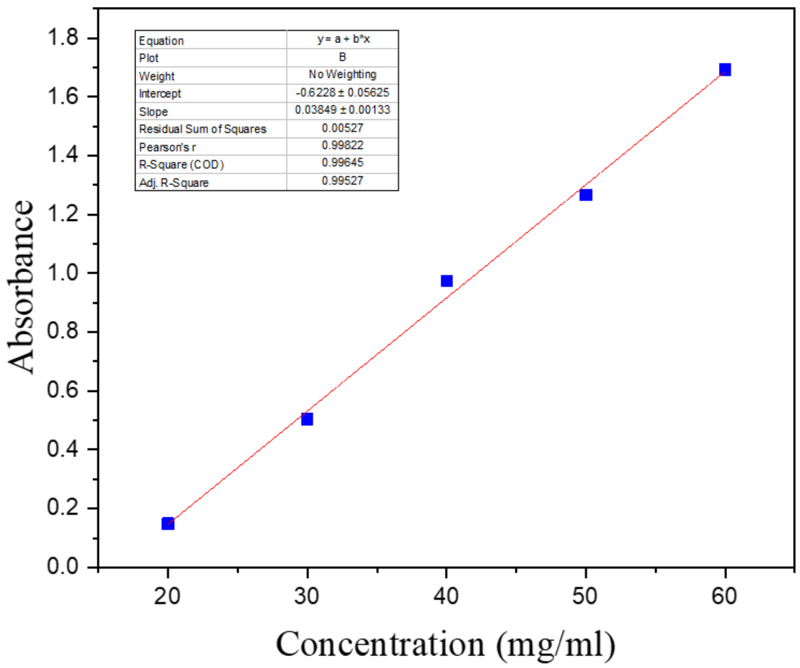
Equation
y= a + b*x
Plot
B
Weight
No Weighting
Intercept
-0.6228 ± 0.05625
Slope
0.03849 ± 0.00133
Residual Sum of Squares
0.00527
Pearson's r
0.99822
R-Square (COD)
0.99645
Adj. R-Square
0.99527
Absorbance
0.0
0.2
0.4
0.6
0.8
1.0
1.2
1.4
1.6
1.8
20
30
40
50
60
Concentration (mg/ml)

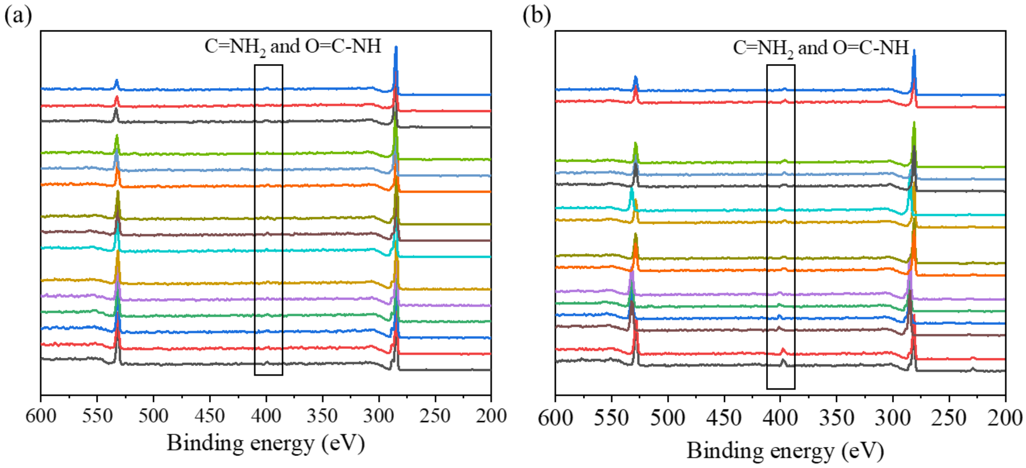

XPS depth of PCL-A (a) and PCL-A-OHAGly-OMe (b)
